# Association between cumulative changes in the Wells score and the risk of stroke-associated pneumonia in patients with acute ischemic stroke: results from the REMISE study

**DOI:** 10.3389/fneur.2025.1709155

**Published:** 2026-01-12

**Authors:** Jing Yu, Dongze Li, Jin Chen, Yi Liu, Wei Zhang, Yan Zhong, Yan Ma, Zhi Zeng, Qinqin Wu, Zhi Wan

**Affiliations:** 1 Department of Emergency Medicine, Rare Diseases Center, National Clinical Research Center for Geriatrics, West China Hospital, West China School of Medicine, Sichuan University, Chengdu, China; 2 College of Arts, Sichuan University, Chengdu, China; 3 Department of Cadre Health Care, National Clinical Research Center for Geriatrics, West China Hospital, West China School of Medicine, Sichuan University, Chengdu, China; 4 School of Public Health, Xinjiang Medical University, Urumqi, China; 5 Health Management Center, General Practice Medical Center, National Clinical Research Center for Geriatrics, West China Hospital, West China School of Medicine, Sichuan University, Chengdu, China

**Keywords:** ischemic stroke, pneumonia severity index, stroke-associated pneumonia, thrombotic burden, Wells score

## Abstract

**Introduction:**

The Wells score, a reliable indicator of thrombotic burden, has been associated with stroke-associated pneumonia (SAP) in patients with acute ischemic stroke (AIS). However, the impact of changes in dynamic Wells scores on the incidence and severity of SAP remains poorly understood.

**Methods:**

A total of 767 participants with AIS were recruited from the Retrospective Multicenter Study for Ischemic Stroke Evaluation. Two Wells score measurements (i.e., at baseline and within the first 3 days) were obtained. Changes in dynamic Wells scores were then estimated using two indices: the cumulative Wells score and dynamic Wells score classes based on K-means analysis. The cumulative Wells score was calculated as the weighted sum of the mean Wells score value for each time interval (value × time). Dynamic Wells score classification was determined using K-means clustering analysis. Logistic regression was employed to analyze the effects of changes in dynamic Wells scores on the incidence of SAP.

**Results:**

Among the included patients, 263 developed SAP. Four dynamic Wells score classes were identified. The incidence of SAP increased significantly with increasing cumulative Wells scores. After adjusting for confounders, an elevated cumulative Wells score was significantly associated with an increased incidence of SAP (*p* < 0.001). Compared with the class 1 group, the class 2, 3, and 4 groups had odds ratios for SAP incidence of 2.01 (*p* = 0.048), 3.71 (*p* = 0.001), and 9.90 (*p* < 0.001), respectively. Adding changes in dynamic Wells scores to a conventional risk model for SAP improved discrimination and calibration. Changes in dynamic Wells scores were positively correlated with the pneumonia severity index.

**Discussion:**

Changes in dynamic Wells scores were independently associated with the incidence of SAP. Therefore, dynamic monitoring of changes in the Wells score may assist in the early identification of patients at high risk of developing SAP.

## 1 Introduction

Stroke-associated pneumonia (SAP) is a critical complication that affects 10–30% of patients hospitalized for acute ischemic stroke (AIS) (1, 2). It significantly increases 30-day mortality rates, prolongs hospitalization, and contributes to long-term disability (3–6). The negative effects of SAP result from neurological deterioration due to hypoxia, systemic inflammatory cascades, and metabolic disturbances that impede recovery (4, 7). Therefore, early identification of patients at high risk of developing SAP is crucial for implementing targeted preventive strategies (7–13).

Contemporary SAP risk models primarily rely on static admission parameters, such as dysphagia, age, and stroke severity (measured using the National Institutes of Health Stroke Scale [NIHSS] or modified Rankin Scale), as exemplified by models such as A2DS^2^ (Age, Atrial fibrillation, Dysphagia, Sex, Stroke Severity) and ISAN (Independence before stroke, Sex, Age, NIHSS) (14–18). Among these two models, A2DS^2^ typically demonstrates superior predictive accuracy (19). Despite the clinical accessibility of these tools, they have two fundamental limitations: a lack of temporal flexibility in capturing the evolving pathophysiology and the exclusion of thromboinflammatory mechanisms central to the pathogenesis of SAP (1, 20). Poststroke immunothrombosis induces systemic hypercoagulability and microvascular dysfunction (21–23). Our previous studies substantiate this association by demonstrating that elevated thrombotic biomarkers are strong predictors of SAP risk (1, 24, 25). However, biomarker-based approaches are constrained by delayed laboratory processing and limited feasibility for serial assessment. Hence, no current tool allows rapid, bedside-compatible quantification of dynamic thrombotic burden for early SAP risk mitigation.

The Wells score is a pragmatic clinical tool endorsed by international guidelines for evaluating thrombotic risk (26–28). It provides a rapid bedside assessment of systemic thrombotic burden. Our preliminary data indicate its relevance in assessing the risk of SAP, with a one-unit increase in the Wells score corresponding to a 2.14-fold increase in the risk of SAP. The Wells score has traditionally been measured at a single time point, without exploration of its temporal fluctuations or the long-term impact of these changes. Thus, evaluating the Wells score dynamically, rather than relying on a one-time measurement, may provide more meaningful prognostic insights. However, few studies have examined the association between longitudinal cumulative changes in the Wells score and the risk of stroke.

Therefore, this study aimed to evaluate dynamic changes in thrombotic burden using changes in dynamic Wells scores, including two indices: the cumulative Wells score, which integrates serial measurements over time, and the dynamic Wells score class determined through K-means analysis, which describes distinct trajectory phenotypes in patients with AIS. Thereafter, the associations between changes in the dynamic Wells score and the risk of SAP, as well as between changes in the dynamic Wells score and the severity of SAP, were analyzed.

## 2 Methods

### 2.1 Study population

This *post-hoc* analysis of a retrospective multicenter study used data from the Retrospective Multicenter Study for Ischemic Stroke Evaluation (REMISE), which enrolled patients with AIS between January 2020 and December 2020 who were admitted to the emergency departments (EDs) of five grade A tertiary hospitals across China. Details regarding the rationale and design of the REMISE study have been reported previously (1, 24, 25, 29). The trial was registered with the Chinese Clinical Trial Registry (www.chictr.org.cn; Identifier: ChiCTR2100052025). This study was conducted in accordance with the ethical principles of the Declaration of Helsinki and was approved by the Human Ethics Committee of Sichuan University West China Hospital and the ethics committees of the other participating hospitals.

Participants who met the following criteria were included in this study: (1) age of ≥18 years; (2) first-time diagnosis of AIS; (3) < 12 h between symptom onset and ED admission; (4) Wells score calculated within 1 h of admission and repeatedly within the first 3 days after admission; (5) no history of pneumonia; and (6) complete medical records available for review. The exclusion criteria were as follows: a diagnosis of hemorrhage or transient ischemic attack; presence of malignant tumors; severe liver or kidney dysfunction; newly diagnosed SAP before dynamic Wells score assessment; a history of clinical signs of infection within 30 days before or at the onset of AIS; receipt of prophylactic antibiotics before admission or during initial evaluation; or known inflammatory or autoimmune disorders.

### 2.2 Data collection

Clinical data were collected from the electronic medical records of each participating hospital using standard case report forms from the REMISE database. The data included demographic characteristics, vital signs, laboratory results, imaging findings, inpatient complications, adverse outcomes, and treatment details during hospitalization. All data were anonymized to ensure confidentiality and compliance with ethical standards. The Wells score, which ranges from 0 to 11 points, incorporates risk factors, clinical signs, and the presence or absence of alternative diagnoses (30). The NIHSS was used to assess stroke-related neurological deficits at admission and discharge, with higher scores indicating more severe impairment (31). The pneumonia severity index was applied to assess pneumonia severity and includes 20 demographic and clinical variables (32). Furthermore, the A2DS^2^ score, a screening tool for SAP, was calculated based on age, dysphagia, male sex, atrial fibrillation, and stroke severity (20).

### 2.3 Calculation of cumulative changes in the Wells score

The method for calculating the cumulative index has been described previously (33, 34). The cumulative Wells score was defined as the sum of the average Wells score for each pair of consecutive examinations multiplied by the time interval between assessments within the first 3 days after admission. As an area-under-the-curve estimate (mean Wells score × time span), the cumulative Wells score was calculated as follows: (Wells score at admission + Wells score within 3 days of admission)/2 × the time interval between the two evaluations. Participants were then stratified into low-risk (score = 0, reference group), moderate-risk (scores 1–12), and high-risk (scores >13) groups according to cumulative Wells score tertiles.

Subsequently, K-means clustering was used to stratify patients into different Wells score trajectory groups based on their dynamic Wells scores.

### 2.4 Outcome and follow-up

The primary outcome was SAP, which was confirmed within 7 days after stroke onset through a combination of hospital medical records, telephone contact with family members, and death registration at the Sichuan Provincial Center for Disease Control and Prevention. Additionally, SAP was diagnosed according to the 2019 American Heart Association stroke guidelines (35). In this study, the median time to SAP diagnosis was 4 (2–6) days after admission.

### 2.5 Statistical analyses

Normally distributed continuous clinical variables were presented as mean ± standard deviation, non-normally distributed continuous variables were expressed as median (25th–75th percentiles), and categorical variables were expressed as frequencies and percentages. Missing data for covariates (<2%) were imputed using multiple imputation. Parametric and nonparametric patient characteristics were compared using one-way analysis of variance and the Kruskal–Wallis H test, respectively. Categorical variables were compared using the chi-square test or Fisher’s exact test.

K-means clustering was used to stratify patients into different groups according to their dynamic Wells scores. Logistic regression analyses were performed to explore the association between changes in dynamic Wells scores and SAP by calculating odds ratios (ORs) and 95% confidence intervals (CIs) across four models. Model 1 was a crude model with no adjustments. Model 2 was adjusted for age, sex, smoking status, drinking status, body mass index (BMI), and dysphagia. Model 3 was adjusted for the variables in Model 2, along with hypertension, diabetes, hyperlipidemia, and atrial fibrillation. Model 4 was adjusted for the variables in Model 3, along with white blood cell (WBC) count, platelet count, D-dimer level, estimated glomerular filtration rate, and stroke severity (NIHSS score).

The C-statistic was calculated to assess the predictive performance of changes in dynamic Wells scores for SAP incidence. The incremental predictive value of changes in dynamic Wells scores was evaluated using the integrated discrimination improvement (IDI) and net reclassification index (NRI) (36). Additionally, decision curve analysis (DCA) was employed to illustrate the clinical utility of changes in dynamic Wells scores (37). Model calibration was assessed using the Hosmer–Lemeshow test (38).

Subgroup analyses were conducted to examine whether demographic and health-related variables, including age, sex, drinking status, smoking status, hypertension, diabetes, hyperlipidemia, atrial fibrillation, WBC count, and NIHSS score, modified the association between changes in dynamic Wells scores and SAP. *p* values for interaction were assessed using likelihood ratio tests and interaction terms.

All reported *p* values were two-tailed. All analyses were conducted using SPSS version 26.0 (IBM Corp., Armonk, NY, United States) and R software version 4.5.1 (R Foundation for Statistical Computing, Vienna, Austria), with *p* < 0.05 indicating statistical significance.

## 3 Results

### 3.1 Demographic and clinical characteristics of patients with AIS

**Figure 1:**
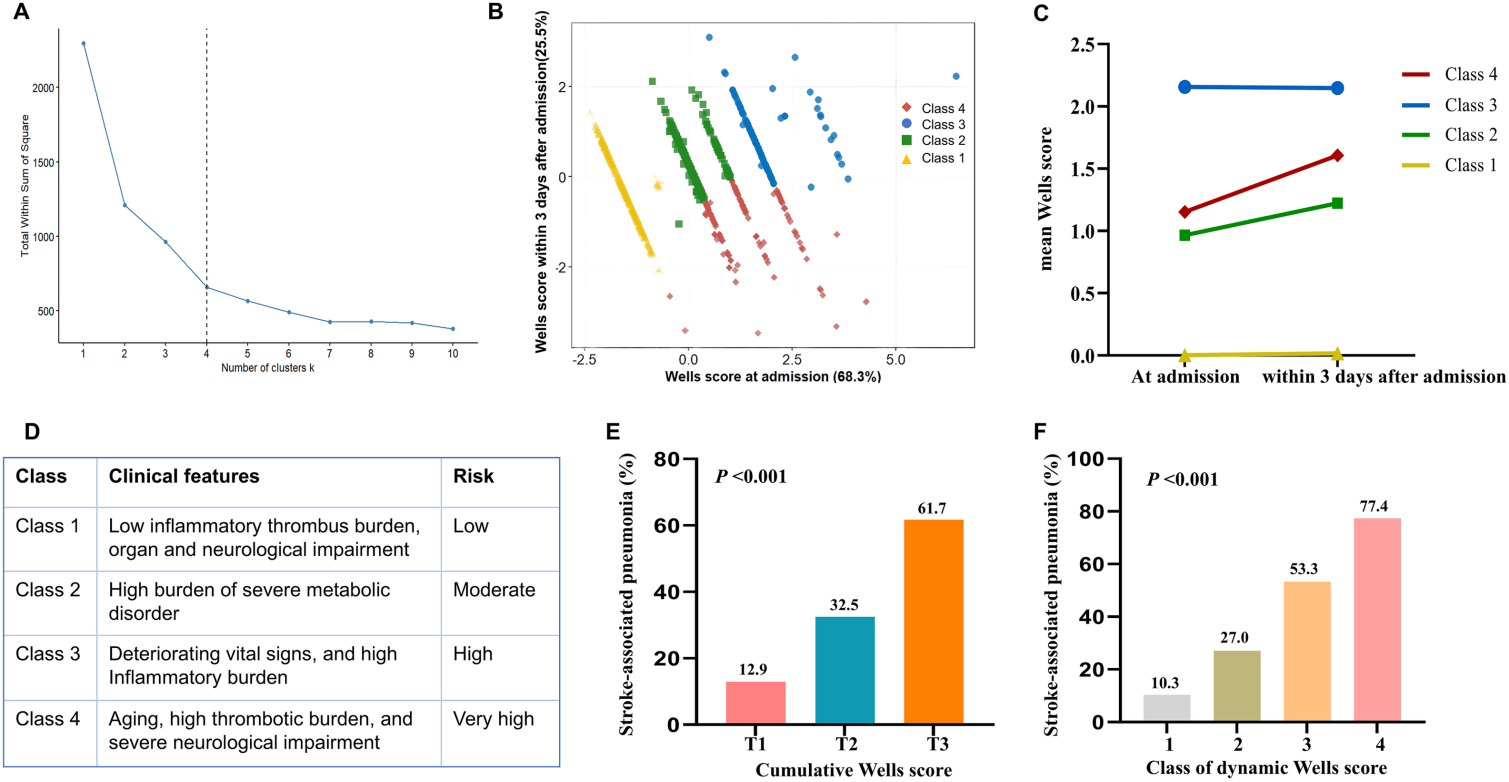
**(A)** *K*-means clustering method for clustering the changes of Wells scores between admission and within 3 days after admission; **(B)** Four clusters were found using the *K*-means method with Euclidean distance; **(C)** Data visualization for different classes of the change in the Wells score; **(D)** Clinical features for patient with different dynamic Wells score risk classes; **(E)** The incidence of stroke-associated pneumonia among patients with different cumulative Wells score tertiles; **(F)** The incidence of stroke-associated pneumonia among patients with different dynamic Wells score risk classes.

Among the 1,050 patients with AIS, 767 were included after applying the eligibility criteria. K-means clustering identified four distinct dynamic Wells score trajectories (Figures 1A–C): (1) class 1: Wells scores varied from 0.003 at admission to 0.018 at the second measurement, indicating persistently low scores with a slight increase (i.e., the stable low-risk group); (2) class 2: Wells scores varied from 0.966 at admission to 1.223 at the second measurement, indicating moderate scores with a moderate increase (i.e., the moderate-risk group); (3) class 3: Wells scores varied from 1.153 at admission to 1.606 at the second measurement, indicating higher scores with a marked increase (i.e., the high-risk group); and (4) class 4: Wells scores varied from 2.156 at admission to 2.148 at the second measurement, indicating the highest scores with no significant decrease (i.e., the persistent very high-risk group).

Table 1 summarizes the baseline characteristics of the four groups based on changes in their Wells scores (classes 1–4). Significant differences in age, male sex, comorbidities (hyperlipidemia and atrial fibrillation), vital signs (dysphagia, respiratory rate, heart rate, body temperature, and BMI), inflammatory biomarkers (red and white blood cell counts), metabolic biomarkers (blood glucose, high-density lipoprotein, low-density lipoprotein [LDL], and triglyceride [TG] levels), organ damage markers (blood urea nitrogen and creatinine levels), thrombosis markers (D-dimer and fibrinogen [FIB] levels), and neurological impairment markers (NIHSS) were observed across the four classes. Figure 1D illustrates the clinical phenotypes of each class. Specifically, class 4 represented aging, high thrombotic load, and severe neurological impairment, with the highest mean age, elevated D-dimer and FIB levels, and significantly higher NIHSS scores. Conversely, class 3 represented younger patients with deteriorating vital signs and a high inflammatory load, with the highest proportions of dysphagia and elevated respiratory rate, heart rate, body temperature, BMI, WBC count, and systemic immune-inflammation index (SII). Class 2 represented low inflammatory and thrombotic burden as well as low organ and neurological impairment, with the fewest comorbidities and inflammatory biomarkers, metabolic biomarkers, organ damage markers, and neurological impairment markers. Class 1 represented a high burden of severe metabolic disorders, with a high incidence of hyperlipidemia and elevated BMI and LDL and TG levels. Supplementary Table 1 shows the baseline characteristics of patients with AIS stratified according to their cumulative Wells scores.

**Table 1:** Baseline characteristics of participants stratified by changes in the Wells score between admission and within 3 days after admission.

Variables	Total (*n* = 767)	Class 1 (*n* = 271)	Class 2 (*n* = 237)	Class 3 (*n* = 122)	Class 4 (*n* = 137)	*p*-value
Age, years	66 ± 14	60 ± 14	64 ± 12	67 ± 13	77 ± 9	<0.001
Male sex, *n* (%)	484 (63.1)	178 (65.7)	151 (63.7)	62 (50.8)	93 (67.9)	0.018
Smoking, *n* (%)	310 (40.4)	123 (45.4)	95 (40.1)	43 (35.3)	49 (35.8)	0.146
Drinking, *n* (%)	229 (29.9)	93 (34.3)	70 (29.5)	31 (25.4)	35 (25.6)	0.175
Hypertension, *n* (%)	467 (60.9)	161 (59.4)	141 (59.5)	80 (65.6)	85 (62.0)	0.649
Diabetes, *n* (%)	191 (24.9)	63 (23.3)	60 (25.3)	30 (24.6)	38 (27.7)	0.798
Hyperlipidemia, *n* (%)	7 (9.5)	27 (10.0)	30 (12.7)	6 (4.9)	8 (5.8)	0.045
Dysphagia, *n* (%)	73 (9.5)	8 (3.0)	18 (7.6)	27 (22.1)	20 (14.6)	<0.001
Atrial fibrillation, *n* (%)	195 (25.4)	33 (12.2)	49 (20.7)	40 (32.8)	73 (53.3)	<0.001
SBP, mmHg	146 ± 25	144 ± 25	146 ± 25	145 ± 24	147 ± 26	0.759
DBP, mmHg	87 ± 16	86 ± 16	87 ± 16	89 ± 15	86 ± 17	0.457
Temperature, °C	36.5 ± 0.3	36.5 ± 0.3	36.5 ± 0.3	36.5 ± 0.4	36.5 ± 0.3	0.013
Respiratory rate, /min	20 ± 2	20 ± 2	19 ± 2	20 ± 3	20 ± 3	<0.001
Heart rate, /min	79 (69–92)	78 (69–89)	80 (69–88)	87 (73–100)	76 (65–91)	0.007
BMI, kg/m^2^	23.0 (19.9–25.5)	23.5 (21.4–25.9)	23.6 (20.1–25.3)	23.0 (17.5–26.6)	20.9 (17.3–23.8)	<0.001
Laboratory findings						
RBC count, ×10^12^/L	4.49 ± 0.63	4.57 ± 0.55	4.56 ± 0.62	4.43 ± 0.73	4.26 ± 0.66	<0.001
WBC count, ×10^9^/L	7.77 ± 2.82	7.06 ± 2.42	7.79 ± 2.69	8.83 ± 3.28	8.19 ± 2.96	<0.001
PLT count, ×10^9^/L	183.62 ± 72.24	186.22 ± 70.58	183.78 ± 71.54	193.07 ± 81.36	169.90 ± 66.75	0.063
SII	644 (380–1,128)	503 (302–775)	679 (409–1,151)	911 (478–1,526)	723 (470–1,588)	<0.001
Blood glucose, mmol/L	7.71 ± 3.14	7.10 ± 2.91	7.90 ± 3.22	8.03 ± 3.68	8.29 ± 2.77	<0.001
HbA1c, %	6.53 ± 1.46	6.47 ± 1.50	6.62 ± 1.53	6.51 ± 1.42	6.52 ± 1.29	0.742
HDL, mmol/L	1.25 ± 0.40	1.28 ± 0.40	1.25 ± 0.39	1.19 ± 0.40	1.26 ± 0.41	0.308
LDL, mmol/L	2.40 (1.88–3.04)	2.37 (1.82–3.12)	2.50 (1.90–3.22)	2.42 (1.95–3.02)	2.34 (1.79–2.82)	0.016
TG, mmol/L	1.23 (0.91–1.85)	1.29 (0.90–1.92)	1.39 (0.85–2.02)	1.35 (0.98–2.08)	1.06 (0.80–1.58)	0.010
AST, U/L	22 (18–28)	21 (17–26)	22 (18–29)	23 (18–29)	22 (18–29)	0.128
ALT, U/L	19 (14–28)	19 (15–29)	19 (14–30)	18 (13–26)	17 (13–26)	0.388
Albumin, g/L	41.57 ± 4.14	43.07 ± 3.22	41.98 ± 3.79	40.07 ± 4.97	39.26 ± 4.07	<0.001
TBL, μmol/L	11.2 (8.60–15.2)	10.70 (8.23–14.60)	11.25 (8.60–15.28)	11.80 (8.90–15.70)	11.95 (8.95–16.38)	0.161
DBL, μmol/L	3.85 (2.90–5.30)	3.70 (2.85–4.80)	3.75 (2.80–5.20)	4.10 (3.00–5.40)	4.30 (3.00–6.40)	0.009
UA, μmol/L	341.59 ± 108.04	340.22 ± 103.02	342.05 ± 102.78	317.69 ± 106.45	364.58 ± 123.23	0.007
BUN, mmol/L	5.60 (4.40–7.01)	5.40 (4.30–6.70)	5.25 (4.23–6.68)	5.50 (4.35–7.40)	6.30 (5.55–8.90)	<0.001
Creatinine, μmol/L	74 (63–88)	72 (60–83)	71 (62–81)	76 (63–90)	80 (68–98)	<0.001
D-dimer, mg/L	0.72 (0.30–1.74)	0.34 (0.19–0.74)	0.59 (0.29–1.22)	1.28 (0.58–4.70)	1.45 (0.76–4.54)	<0.001
FIB, g/L	3.05 ± 1.03	2.84 ± 0.75	2.97 ± 1.00	3.36 ± 1.28	3.37 ± 1.17	<0.001
Risk scores						
NIHSS score	9.41 ± 8.12	3.46 ± 4.62	9.27 ± 6.91	13.28 ± 8.00	14.82 ± 8.12	<0.001
A2DS2 score	4 (2–5)	3 (1–4)	4 (3–5)	4 (4–6)	6 (5–7)	<0.001
Cumulative Wells score	7.0 (0–16.0)	0 (0–0)	9.0 (7.0–13.5)	21.0 (14.8–30.0)	13.50 (6.0–22.0)	<0.001

SBP, systolic blood pressure; DBP, diastolic blood pressure; BMI, body mass index; RBC, red blood cell; WBC, white blood cell; PLT, platelet; SII, systemic immune-inflammation index; HbA1c, glycosylated hemoglobin; HDL, high-density lipoprotein; LDL, low-density lipoprotein; TG, triglyceride; AST, aspartate aminotransferase; ALT, alanine aminotransferase; TBL, total bilirubin; DBL, direct bilirubin; UA, uric acid; BUN, blood urea nitrogen; FIB, fibrinogen; NIHSS, National Institutes of Health Stroke Scale; A2DS2, Age, Atrial fibrillation, Dysphagia, Sex, Stroke Severity.

### 3.2 Association between cumulative changes in the Wells score and risk of SAP

Among the included patients, 263 developed SAP. The incidence of SAP increased substantially with cumulative Wells score tertiles, reaching a maximum incidence of 61.7% in the highest tertile (Figure 1E). After adjustment for confounders (Model 3), higher cumulative Wells scores remained significantly associated with SAP. Compared with the lowest tertile, the moderate and high tertiles demonstrated significantly increased risks (Table 2). When the cumulative Wells score was analyzed as a continuous variable, a 1-unit increase was associated with a 7% higher risk of SAP (OR: 1.07, 95% CI: 1.04–1.09; *p* < 0.001) after full adjustment for potential confounders. These findings indicate that the cumulative Wells score may serve as a predictor of SAP.

**Table 2:** Association between changes in the Wells score between admission and within 3 days after admission and the risk of SAP.

Variables	Model 1		Model 2		Model 3		Model 4	
	OR (95% CI)	*P*-value	OR (95% CI)	*P*-value	OR (95% CI)	*P*-value	OR (95% CI)	*P*-value
Dynamic Wells score class								
		<0.001		<0.001		<0.001		<0.001
Class 1	1		1		1		1	
Class 2	3.21 (1.98–5.22)	<0.001	2.76 (1.63–4.65)	<0.001	2.68 (1.58–4.55)	<0.001	2.01 (1.01–4.01)	0.048
Class 3	9.90 (5.83–16.79)	<0.001	8.70 (4.83–15.67)	<0.001	8.50 (4.65–15.51)	<0.001	3.71 (1.70–8.11)	0.001
Class 4	29.68 (16.96–51.93)	<0.001	19.26 (10.23–36.24)	<0.001	16.50 (8.63–31.52)	<0.001	9.90 (4.24–23.11)	<0.001
Cumulative Wells score								
Per 1-unit increase	1.09 (1.07–1.10)	<0.001	1.08 (1.06–1.10)	<0.001	1.07 (1.05–1.09)	<0.001	1.07 (1.04–1.09)	<0.001
Tertile 1	1	<0.001	1	<0.001	1	<0.001	1	<0.001
Tertile 2	3.27 (2.11–5.07)	<0.001	2.65 (1.64–4.30)	<0.001	2.42 (1.48–3.96)	<0.001	2.20 (1.15–4.19)	0.016
Tertile 3	10.92 (7.05–16.92)	<0.001	8.78 (5.38–14.35)	<0.001	7.94 (4.82–13.09)	<0.001	7.06 (3.77–13.21)	<0.001

Model 1: Unadjusted.

Model 2: Adjusted for age, sex, systolic blood pressure, diastolic blood pressure, smoking status, drinking status, body mass index, and dysphagia.

Model 3: Adjusted for the variables in Model 2 plus hypertension, diabetes, hyperlipidemia, and atrial fibrillation.

Model 4: Adjusted for the variables in Model 3 plus red blood cell count, white blood cell count, platelet count, and D-dimer level.

OR, odds ratio; CI, confidence interval.

Patients with higher dynamic Wells score risk classes had a significantly greater probability of developing SAP than those with lower risk classes (Figure 1F). Compared with patients in class 1, those in classes 2, 3, and 4 had significantly increased risks of SAP, with adjusted ORs of 2.01 (*p* = 0.048), 3.71 (*p* = 0.001), and 9.90 (*p* < 0.001), respectively, demonstrating a clear risk gradient (Table 2). This gradient underscores the prognostic utility of dynamic Wells score classes in stratifying SAP risk.

### 3.3 Subgroup and sensitivity analyses

The association between cumulative changes in the Wells score and the risk of SAP remained robust across various subgroups, reinforcing the predictive utility of the cumulative Wells score and dynamic Wells score classes. No significant interactions were observed between cumulative Wells scores or dynamic Wells score classes and subgroup variables. Furthermore, sensitivity analyses yielded consistent results after excluding individuals with comorbidities (hypertension, diabetes, and hyperlipidemia) (Supplementary Table 2), high SII (>600) (Supplementary Table 3), and severe neurological impairment (NIHSS score >16) (Supplementary Table 4).

### 3.4 Incremental predictive value of cumulative changes in the Wells score

**Figure 2:**
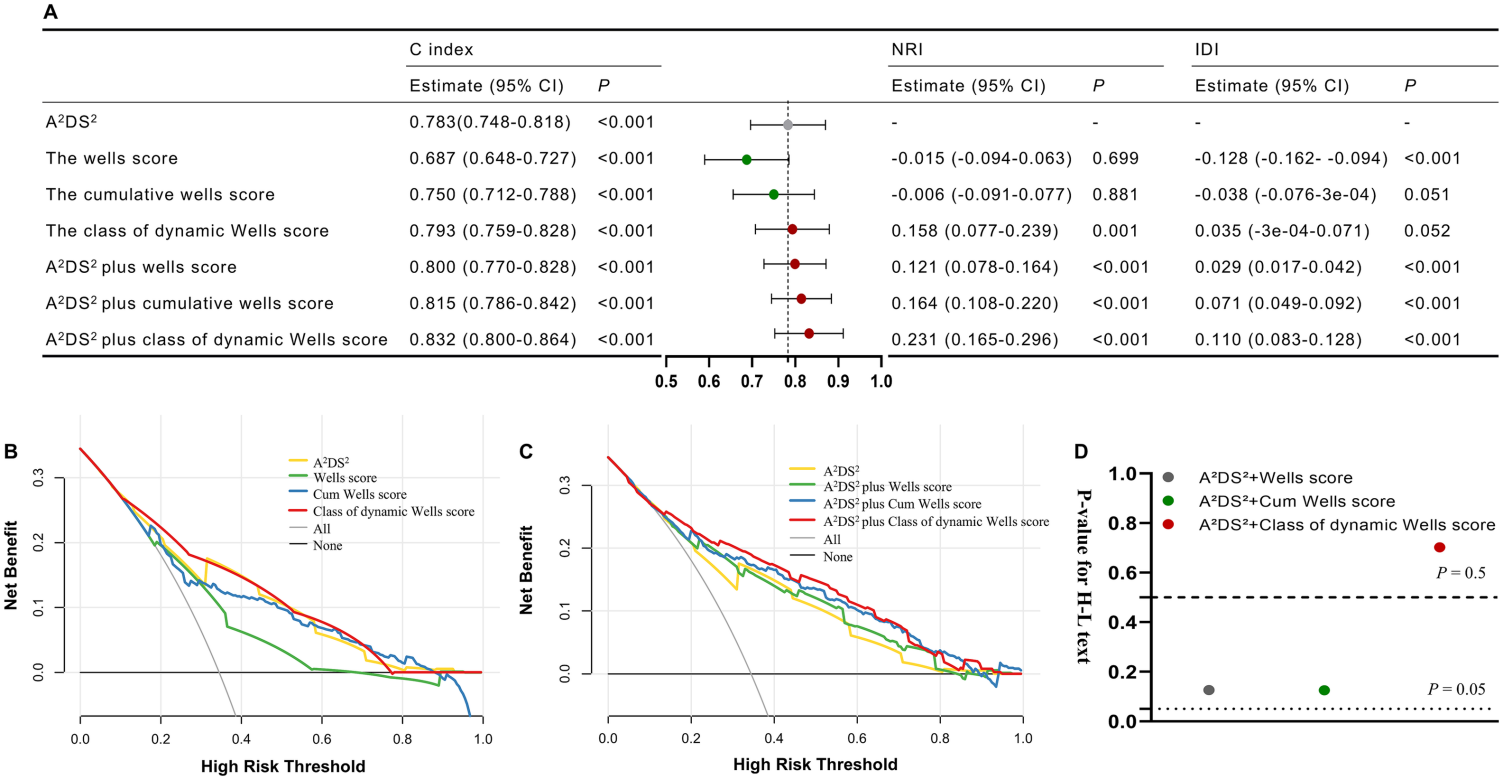
**(A)** The evaluation of discriminative capabilities and incremental predictive value of cumulative changes in the Wells score for by C index, NRI, and IDI; **(B)** The comparison of clinical utility by decision curves for different cumulative changes in the Wells score; **(C)** The comparison of clinical utility through decision curves for combinations of different cumulative changes in the Wells score with the A2DS2 score; **(D)** The *p*-values of the Hosmer–Lemeshow tests for different combinations of different cumulative changes in the Wells score with the A2DS2 score. IDI, integrated discrimination improvement; NRI, net reclassification index; SAP, stroke-associated pneumonia.

To assess the predictive value of cumulative changes in the Wells score for SAP risk, we further analyzed the C-statistic, NRI, and IDI. The C-statistic of the A2DS^2^ score improved significantly with the addition of the Wells score at admission (from 0.783 to 0.800, *p* < 0.001), the cumulative Wells score (from 0.783 to 0.815, *p* < 0.001), and the dynamic Wells score class (from 0.783 to 0.834, *p* < 0.001) (Figure 2A). DCA showed that the net benefit of the dynamic Wells score class was higher than that of the A2DS^2^ score, the Wells score at admission, and the cumulative Wells score across all threshold probabilities (Figure 2B). Additionally, risk reclassification and discriminatory power improved substantially, with NRIs of 12.1, 16.4, and 23.1% (*p* < 0.001) and IDIs of 2.9, 7.1, and 11.0% (*p* < 0.001) for the Wells score at admission, cumulative Wells score, and dynamic Wells score class, respectively (Figure 2A). These findings indicate that incorporating cumulative changes in the Wells score improves prediction efficiency for SAP risk. DCA further demonstrated that combining the dynamic Wells score class with the A2DS^2^ score provided the greatest net benefit compared with any other combination (Figure 2C).

We then evaluated calibration using the Hosmer–Lemeshow test, which showed that the combination of the dynamic Wells score class and the A2DS^2^ score had the best calibration for predicting SAP risk (Figure 2D).

### 3.5 Relationship between cumulative changes in the Wells score and pneumonia severity

**Figure 3:**
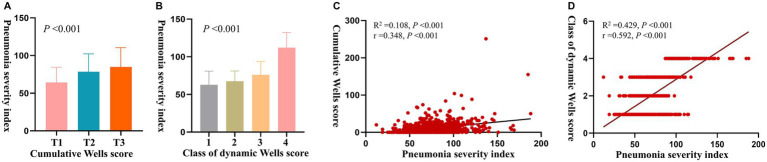
**(A)** Pneumonia severity indexes among patients with different cumulative Wells score tertiles; **(B)** pneumonia severity indexes among patients with different dynamic Wells score risk classes; **(C)** spearman’s correlation analysis of the pneumonia severity index and the cumulative Wells score; **(D)** spearman’s correlation analysis of the pneumonia severity index and dynamic Wells score risk classes.

Patients with higher cumulative Wells scores (Figure 3A) and higher dynamic Wells score classes had greater pneumonia severity index values (Figure 3B). Spearman’s correlation analysis showed that the pneumonia severity index was positively associated with the cumulative Wells score (Figure 3C) and dynamic Wells score class (Figure 3D). Among these measures, the dynamic Wells score class exhibited the strongest correlation with pneumonia severity, indicating superior predictive ability for pneumonia severity.

## 4 Discussion

Our study provides valuable insights into the association between dynamic changes in thrombotic burden (evaluated using two indices, namely the cumulative Wells score and dynamic Wells score class) and both the incidence and severity of SAP among patients with AIS. Our findings revealed that patients in dynamic Wells score class 4 (the highest Wells score without a significant decrease) exhibited a significantly higher risk of SAP than those in other classes. Furthermore, the cumulative Wells score was independently associated with an increased risk of SAP, with patients in higher cumulative Wells score tertiles demonstrating progressively greater SAP risk than those in lower tertiles. The calibration and discrimination of models combining cumulative changes in the Wells score with the A2DS^2^ score were significantly superior to those of the A2DS^2^ score alone, which is recommended by current guidelines for predicting SAP risk. Moreover, we found that the cumulative Wells score and dynamic Wells score class were each associated with pneumonia severity. Therefore, assessment of dynamic thrombotic burden may provide more accurate stratification of SAP risk, thereby enhancing clinical decision-making and potentially guiding targeted interventions for SAP.

SAP is an extremely common and serious complication of AIS, with incidence rates ranging from 7 to 38% (4, 14, 25). Unfortunately, recent advancements in the prevention of SAP have been limited. Some clinical studies investigating the use of preventive antibiotics for SAP have not yielded positive results, despite success in animal research (11, 12). Although comprehensive SAP risk control (CSRC) in the early phase of AIS onset may be an efficient precaution, achieving ideal preventive effects of CSRC based solely on neurological impairment is difficult (39). Therefore, integrating additional novel dimensions into CSRC may be necessary. Identifying novel dimensions that are significantly associated with SAP risk could enable the development of more personalized treatment plans and ultimately reduce the incidence and severity of SAP in patients with AIS. Attempts have been made to determine SAP risk in patients with AIS using various risk variables and predictive scores, such as age, sex, dysphagia, atrial fibrillation, the Glasgow Coma Scale score, and stroke severity, among others (2, 3, 14, 18–20). Based on these dimensions, several scoring systems, including A2DS^2^, ISAN, and ITEM (Infection after sTrokE Model) scores, have been developed to predict SAP risk (3, 15–20). These scoring systems mainly consider aging, stroke etiology, and neurological impairment. However, their predictive power remains suboptimal because of limited sensitivity and specificity (3, 15).

Recent studies have reported that incorporating novel biomarkers, such as inflammatory markers including WBC count; neutrophil-to-lymphocyte ratio; and C-reactive protein, procalcitonin, and interleukin levels, may improve the accuracy of SAP prediction when combined with clinical scoring systems (40–43). Our previous studies have demonstrated that elevated levels of thrombotic biomarkers, such as D-dimer, FIB, and platelet count, are associated with an increased risk of SAP in patients with AIS, indicating that thrombotic burden may play an important role in predicting SAP risk (1). However, evaluation of thrombotic burden has primarily focused on individual or multiple thrombotic biomarkers. Therefore, early assessment of thrombotic burden cannot be fully achieved, and dynamic monitoring of thrombotic changes during the acute phase of AIS remains understudied. Our previous study, which evaluated thrombotic burden using the Wells score (a widely available clinical tool), found that it was independently associated with SAP risk in patients with AIS. Nevertheless, the dynamic changes in thrombotic burden over time and their correlation with the incidence and severity of SAP are yet to be fully elucidated. In the present study, we evaluated dynamic changes in the Wells score using the cumulative Wells score and dynamic Wells score class and found that both indices were independently associated with the incidence and severity of SAP. Moreover, the dynamic Wells score class provided more refined risk stratification, enabling improved prediction of SAP in patients with AIS. This finding underscores the importance of ongoing assessment of thrombotic burden rather than reliance on a single initial evaluation. Collectively, these observations highlight the value of serial assessment of the dynamic Wells score for risk stratification and guidance of timely interventions. Integrating this scoring system into routine clinical practice may enhance prognostic accuracy and improve patient management.

The reasons underlying the association between the Wells score and SAP remain incompletely understood. Our preliminary data suggest that a high thrombotic burden, as reflected by elevated levels of thrombotic biomarkers, may predispose patients with AIS to SAP, indicating a potential link between increased thrombotic load and the incidence and progression of SAP (1, 24, 25). Fluctuations in the Wells score may reflect evolving thrombotic activity or inflammatory responses following AIS, which could directly influence the development of SAP. Moreover, the ability of the dynamic Wells score to capture changes in thrombotic burden over time highlights its potential utility in guiding personalized treatment strategies during the early phases of AIS. Furthermore, after categorizing patients into four groups based on their dynamic Wells score trajectories, the present study identified distinct patterns of clinical outcomes among these groups. Based on our findings, the dynamic Wells score has pathophysiological implications related to aging, metabolic disorders, deteriorating vital signs, inflammatory burden, thrombus formation, and organ and neurological impairment. The high-risk dynamic Wells score class (class 4) was significantly associated with advanced age, high thrombotic load, and severe neurological impairment. These findings suggest that changes in the dynamic Wells score are a preferred index because of their simplicity and ease of acquisition for comprehensive multidimensional assessment. Therefore, dynamic Wells score changes may serve as a valuable tool for risk stratification and clinical decision-making in patients with AIS.

The strengths of this study include the use of a multicenter representative cohort and the evaluation of dynamic changes in the Wells score, which provide a more comprehensive understanding of its predictive value for SAP in patients with AIS. Furthermore, to the best of our knowledge, this study is the first to investigate the association between dynamic Wells score changes and the risk of SAP.

Nevertheless, this study has several limitations. First, the retrospective *post hoc* design introduces potential selection and recall biases. Although we adjusted for known confounders using multivariable logistic regression, residual bias inherent to this study design cannot be excluded. More importantly, the observational nature of the analysis precludes the establishment of causal relationships between changes in dynamic Wells scores and the risk of SAP. Second, SAP was ascertained based on physician diagnoses and medical records, which may have led to misclassification bias. Third, dynamic Wells scores were assessed at only two time points; more frequent assessments would allow a more precise characterization of changes in thrombotic burden over time. Finally, multiple comparisons were performed without adjustment for multiplicity, which may have increased the risk of type I error.

## 5 Conclusion

This study evaluated dynamic changes in thrombotic burden among patients with AIS using two indices: the cumulative Wells score and dynamic Wells score classes derived from K-means analysis. Accordingly, distinct patterns of dynamic thrombotic burden were identified and found to be associated with the risk of SAP. Progression of thrombotic burden was associated with an increased risk of incident SAP, whereas recovery of thrombotic burden was associated with a decreased risk of incident SAP. Future research should include prospective validation, incorporation of stroke-specific inflammatory biomarkers, and integration of dynamic physiological monitoring to improve the translational applicability of this approach. Additionally, precise preventive strategies to delay thrombotic burden progression as well as tailored interventions to reverse thrombotic burden should be developed to improve the management of AIS.

## Data Availability

The raw data supporting the conclusions of this article will be made available by the authors, without undue reservation.
