# Supplementary material for: Association between cumulative changes in the Wells score and the risk of stroke-associated pneumonia in patients with acute ischemic stroke: results from the REMISE study
Source: Front Neurol. 2026 Jan 12;16:1709155. doi: 10.3389/fneur.2025.1709155 (PMC12832495; doi:10.3389/fneur.2025.1709155)
Supplement: Supplementary file 1 [file Table_1.docx]

**Supplementary Materials**

**Association between cumulative changes in the Wells score and the risk of stroke-associated pneumonia in patients with acute ischemic stroke:**

**Results from the REMISE study**

[Supplementary table 1. Baseline characteristics of participants stratified by the cumulative Wells score 1](#_Toc12061)

[Supplementary table 2. Logistic regression results for the association between the changes of the dynamic Wells score and stroke-associated pneumonia among patients without hypertension, diabetes mellitus, and hyperlipidemia. 5](#_Toc14400)

[Supplementary table 3. Logistic regression results for the association between the changes of the dynamic Wells score and stroke-associated pneumonia among patients with low levels of systemic immunoinflammatory status (SII < 600) 7](#_Toc22705)

[Supplementary table 4. Logistic regression results for the association between the changes of the dynamic Wells score and stroke-associated pneumonia among patients with low levels of neurological impairment (NIHSS <16) 9](#_Toc8516)

[Supplementary table 5. Subgroup analysis of the association between the class of dynamic Wells score with stroke-associated pneumonia 11](#_Toc29064)

[Supplementary table 6. Subgroup analysis of the association between the cumulative Wells score with stroke-associated pneumonia 15](#_Toc19379)

Supplementary table 1. Baseline characteristics of participants stratified by the cumulative Wells score

| Variables | Total (n = 767) | T 1 (n = 280) | T 2 (n = 252) | T 3 (n = 235) | *P-value* |
| --- | --- | --- | --- | --- | --- |
|  |  |  |  |  |  |
|  |  |  |  |  |  |
| Age, years | 66 ± 14 | 61 ± 15 | 67 ± 13 | 70 ± 13 | <0.001 |
| Males, n (%) | 484 (63.10) | 185 (66.1) | 156 (61.9) | 143 (60.9) | 0.422 |
| Smoking, n (%) | 310 (40.4) | 128 (45.7) | 96 (38.1) | 86 (36.6) | 0.072 |
| Drinking, n (%) | 229 (29.9) | 98 (35.0) | 67 (26.6) | 64 (27.2) | 0.601 |
| Hypertension, n (%) | 467 (60.9) | 169 (60.4) | 152 (60.3) | 146 (62.1) | 0.896 |
| Diabetes, n (%) | 191 (24.9) | 64 (22.9) | 70 (27.8) | 57 (24.3) | 0.408 |
| Hyperlipidemia, n (%) | 7 (9.5) | 28 (10.0) | 31 (12.3) | 12 (5.1) | 0.020 |
| Dysphagia, n (%) | 73 (9.5) | 12 (4.3) | 19 (7.5) | 42 (17.9) | <0.001 |
| Atrial fibrillation, n (%) | 195 (25.4) | 35 (12.5) | 67 (26.6) | 93 (39.6) | <0.001 |
| SBP, mmHg | 146 ± 25 | 144 ± 25 | 147 ± 25 | 146 ± 26 | 0.550 |
| DBP, mmHg | 87 ± 16 | 86 ± 16 | 87 ± 16 | 87 ± 17 | 0.773 |
| T,℃ | 36.5 ± 0.3 | 36.5 ± 0.3 | 36.5 ± 0.3 | 36.5 ± 0.3 | 0.006 |
| Breath, /min | 20 ± 2 | 20 ± 2 | 20 ± 2 | 20 ± 3 | 0.066 |
| Heart rate, /min | 82 ± 25 | 81 ± 16 | 82 ± 18 | 84 ± 37 | 0.415 |
| BMI, Kg/cm^2^ | 22.7 (19.5 - 25.3) | 25.7 (20.3 - 25.7) | 22.9 (18.8 - 25.1) | 22.4 (17.8 - 25.4) | <0.001 |
| RBC, 10^12^/L | 4.49 ± 0.63 | 4.55 ± 0.59 | 4.48 ± 0.60 | 4.43 ± 0.70 | 0.105 |
| WBC, 10^9^/L | 7.77 ± 2.82 | 7.11 ± 2.43 | 8.01 ± 2.99 | 8.27 ± 2.92 | <0.001 |
| PLT, 10^9^/L | 183.62 ± 72.24 | 186.76 ± 70.52 | 185.02 ± 76.72 | 178.41 ± 69.28 | 0.402 |
| SII | 715 (392 - 1304) | 537 (348 - 782) | 814 (4230 - 1435) | 836 (432 - 1468) | <0.001 |
| Blood glucose, mmol/L | 7.71 ± 3.14 | 7.06 ± 2.87 | 8.16 ± 3.20 | 8.00 ± 3.29 | <0.001 |
| HbA1c, % | 6.53 ± 1.46 | 6.44 ± 1.51 | 6.66 ± 1.49 | 6.51 ± 1.38 | 0.280 |
| HDL, mmol/L | 1.25 ± 0.40 | 1.28 ± 0.40 | 1.21 ± 0.38 | 1.25 ± 0.41 | 0.158 |
| LDL, mmol/L | 2.49 (1.90 - 3.13) | 2.58 (1.86 - 3.18) | 2.38 (1.91 - 3.13) | 2.48 (2.00 - 3.02) | 0.131 |
| TG, mmol/L | 1.22 (0.88 - 1.93) | 1.45 (0.88 - 2.10) | 1.30 (0.95 - 2.09) | 1.14 (0.88 - 1.56) | 0.009 |
| AST, U/L | 22 (18- 28) | 21 (17 - 26) | 23(18 - 31) | 22 (18 - 29) | 0.202 |
| ALT, U/L | 18 (14 - 27) | 20 (15 - 27) | 19 (13 - 28) | 18 (14 - 27) | 0.750 |
| Alb, g/L | 41.57 ± 4.14 | 42.80 ± 3.64 | 41.47 ± 3.95 | 40.23 ± 4.45 | <0.001 |
| TBL, µmol/L | 10.60 (7.98 - 14.90) | 9.95 (7.50 - 143.90) | 11.60 (8.60 - 16.00) | 11.25 (8.60 - 16.13) | 0.031 |
| DBL,µmol/L | 3.70 (2.60 - 5.20) | 3.40 (2.60 - 4.60) | 3.90 (2.90 - 5.50) | 3.95 (2.80 - 5.50) | 0.009 |
| UA, µmol/L | 341.59 ± 108.04 | 338.20 ± 102.21 | 347.26 ± 110.35 | 339.60 ± 112.48 | 0.598 |
| BUN, mmol/L | 5.75 (4.58 - 7.20) | 5.50 (4.40 - 6.50) | 5.70 (4.30 - 7.30) | 6.05 (4.88 - 8.03) | 0.003 |
| Creatinine, µmol/L | 73 (62 - 90) | 72 (62 - 86) | 73 (63 - 91) | 77 (64 - 92) | 0.338 |
| D-dimer, mg/L | 0.82 (0.35 - 2.05) | 0.40 (0.23 - 0.88) | 0.95 (0.37 - 2.57) | 1.37 (0.65 - 3.5) | <0.001 |
| FIB, g/L | 3.05 ± 1.03 | 2.88 ± 0.80 | 3.02 ± 1.02 | 3.311 ± 1.22 | <0.001 |
| NIHSS score | 8 (2 - 14) | 2 (1 - 4) | 8 (3 - 14) | 14 (9 - 18) | <0.001 |
| A^2^DS^2^ scoure | 4 (1 - 6) | 1 (1 - 2) | 4 (2 - 6) | 5 (4 - 7) | <0.001 |
| Wells score | 1 (0 - 1) | 0 (0 - 0) | 1 (1 - 1) | 2 (2 - 2) | <0.001 |

SBP, systolic blood pressure; DBP, diastolic blood pressure; T, temperature; BMI, body mass index; RBC, red blood cell count;WBC, white blood cell count; PLT, platelet count; SII, systemic immunoinflammatory index; HbA1c, glycosylated hemoglobin；HDL, high-density lipoprotein; LDL, low-density lipoprotein; TG, triglycerides; AST, aspartate aminotransferase; ALT, alanine aminotransferase; Alb, albumin; TBL, total bilirubin; DBL, direct bilirubin; UA, uric acid; BUN, blood urea nitrogen; FIB, fibrinogen; NIHSS National Institute of Health Stroke Scale; PSI, pneumonia severity index.

Supplementary table 2. Logistic regression results for the association between the changes of the dynamic Wells score and stroke-associated pneumonia among patients without hypertension, diabetes mellitus, and hyperlipidemia.

| Variables | Model 1 | |  | Model 2 | |  | Model 3 | |  | Model 4 | |
| --- | --- | --- | --- | --- | --- | --- | --- | --- | --- | --- | --- |
|  | OR (95% CI) | *P*-value |  | OR (95% CI) | *P*-value |  | OR (95% CI) | *P*-value |  | OR (95% CI) | *P*-value |
| The class of dynamic Wells score | | | | | | | | | | | |
|  |  | <0.001 |  |  | <0.001 |  |  | <0.001 |  |  | <0.001 |
| Class 1 | 1 |  |  | 1 |  |  | 1 |  |  | 1 |  |
| Class 2 | 4.46 (1.92 - 10.35) | <0.001 |  | 3.29 (1.33 - 8.17) | 0.010 |  | 3.22 (1.29 - 8.04) | 0.012 |  | 3.17 (1.02 - 9.84) | 0.047 |
| Class 3 | 17.97 (6.47 - 49.91) | <0.001 |  | 19.46 (6.20 - 61.15) | <0.001 |  | 18.31 (5.84 - 57.48) | <0.001 |  | 12.35 (3.03 - 50.30) | <0.001 |
| Class 4 | 23.33 (8.79 - 61.97) | <0.001 |  | 18.65 (6.02 - 57.78) | <0.001 |  | 15.40 (4.86 - 48.80) | <0.001 |  | 15.86 (3.54 - 71.02) | <0.001 |
| The cumulative Wells score | | | | | | | | | | | |
| Per 1-unit |  | <0.001 |  | 1.08 (1.04 - 1.11) | <0.001 |  | 1.07 (1.04 - 1.11) | <0.001 |  | 1.06 (1.02 - 1.10) | 0.001 |
| T 1 | 1 |  |  |  | <0.001 |  |  | <0.001 |  |  | <0.001 |
| T 2 | 5.02 (2.285 - 11.21) | <0.001 |  | 3.78 (1.56 - 9.20) | 0.003 |  | 3.43 (1.40 - 8.44) | 0.007 |  | 2.79 (0.85 - 9.14) | 0.091 |
| T 3 | 15.07 (6.58 - 34.50) | <0.001 |  | 12.31 (4.97 - 30.50) | <0.001 |  | 11.46 (4.59 - 28.63) | <0.001 |  | 9.18 (3.03 - 27.78) | <0.001 |

**Model 1** Unadjusted.

**Model 2** adjusted for age, sex, systolic blood pressure, diastolic blood pressure, smoking, drinking, body mass index, dysphagia.

**Model 3** adjusted for age, sex, systolic blood pressure, diastolic blood pressure, smoking, drinking, body mass index, dysphagiax, atrial fibrillation.

**Model 4** adjusted for age, sex, systolic blood pressure, diastolic blood pressure, smoking, drinking, body mass index, dysphagiax, atrial fibrillation, red blood cell count, white blood cell count, platelet count, D-dimer.

OR, odds ratio; CI, confidence interval.

Supplementary table 3. Logistic regression results for the association between the changes of the dynamic Wells score and stroke-associated pneumonia among patients with low levels of systemic immunoinflammatory status (SII < 600)

| Variables | Model 1 | |  | Model 2 | |  | Model 3 | |  | Model 4 | |
| --- | --- | --- | --- | --- | --- | --- | --- | --- | --- | --- | --- |
|  | OR (95% CI) | *P*-value |  | OR (95% CI) | *P*-value |  | OR (95% CI) | *P*-value |  | OR (95% CI) | *P*-value |
| The class of dynamic Wells score | | | | | | | | | | | |
|  |  | <0.001 |  |  | <0.001 |  |  | <0.001 |  |  | <0.001 |
| Class 1 | 1 |  |  | 1 |  |  |  |  |  | 1 |  |
| Class 2 | 2.72 (1.14 - 6.44) | 0.024 |  | 1.93 (0.71 - 5.27) | 0.198 |  | 1.91 (0.69 - 5.32) | 0.213 |  | 2.52 (0.65 - 9.72) | 0.181 |
| Class 3 | 7.72 (2.91 - 20.51) | <0.001 |  | 8.24 (2.75 - 24.74) | <0.001 |  | 7.88 (2.56 - 24.29) | <0.001 |  | 4.08 (0.82 - 19.83) | 0.087 |
| Class 4 | 68.63 (26.72 - 176.25) | <0.001 |  | 51.15 (15.48 - 169.04) | <0.001 |  | 44.78 (13.27 - 151.13) | <0.001 |  | 97.09 (17.62 - 535.16) | <0.001 |
| The cumulative Wells score | | | | | | | | | | | |
| Per 1-unit |  | <0.001 |  | 1.09 (1.06 - 1.13) | <0.001 |  | 1.09 (1.05 - 1.12) | <0.001 |  | 1.08 (1.04 - 1.12) | <0.001 |
| T 1 | 1 |  |  |  | <0.001 |  |  | <0.001 |  |  | <0.001 |
| T 2 | 3.31 (1.53 - 7.16) | 0.002 |  | 2.36 (0.97 - 5.76) | 0.060 |  | 2.11 (0.84 - 5.30) | 0.114 |  | 2.43 (0.72 - 8.25) | 0.154 |
| T 3 | 18.20 (8.61 - 38.47) | <0.001 |  | 13.57 (5.58 - 33.04) | <0.001 |  | 12.82 (5.13 - 32.09) | <0.001 |  | 12.24 (3.88 - 38.61) | <0.001 |

**Model 1** Unadjusted

**Model 2** adjusted for age, sex, systolic blood pressure, diastolic blood pressure, smoking, drinking, body mass index, dysphagia.

**Model 3** adjusted for age, sex, systolic blood pressure, diastolic blood pressure, smoking, drinking, body mass index, dysphagiax, hypertension, diabetes, hyperlipidemia, atrial fibrillation.

**Model 4** adjusted for age, sex, systolic blood pressure, diastolic blood pressure, smoking, drinking, body mass index, dysphagia, hypertension, diabetes, hyperlipidemia, atrial fibrillation, red blood cell count, white blood cell count, platelet count, D-dimer.

SII, systemic immunoinflammatory index; OR, odds ratio; CI, confidence interval.

Supplementary table 4. Logistic regression results for the association between the changes of the dynamic Wells score and stroke-associated pneumonia among patients with low levels of neurological impairment (NIHSS <16)

| Variables | Model 1 | |  | Model 2 | |  | Model 3 | |  | Model 4 | |
| --- | --- | --- | --- | --- | --- | --- | --- | --- | --- | --- | --- |
|  | OR (95% CI) | *P*-value |  | OR (95% CI) | *P*-value |  | OR (95% CI) | *P*-value |  | OR (95% CI) | *P*-value |
| The class of dynamic Wells score | | | | | | | | | | | |
|  |  | <0.001 |  |  | <0.001 |  |  | <0.001 |  |  | <0.001 |
| Class 1 | 1 |  |  | 1 |  |  | 1 |  |  | 1 |  |
| Class 2 | 2.57(1.54 - 4.30) | <0.001 |  | 2.37 (1.37 - 4.13) | 0.002 |  | 2.39 (1.37 - 4.18) | 0.002 |  | 2.02 (0.99 - 4.12) | 0.053 |
| Class 3 | 6.70 (3.75 - 11.96) | <0.001 |  | 6.29 (3.31 - 11.95) | <0.001 |  | 6.13 (3.18 - 11.82) | <0.001 |  | 3.42 (1.52 - 7.70) | 0.003 |
| Class 4 | 19.39 (10.45 - 35.98) | <0.001 |  | 12.85 (6.38 - 25.89) | <0.001 |  | 11.55 (5.60 - 23.81) | <0.001 |  | 10.21 (4.20 - 24.82) | <0.001 |
| The cumulative Wells score | | | | | | | | | | | |
| Per 1-unit |  | <0.001 |  | 1.07 (1.04 - 1.09) | <0.001 |  | 1.07 (1.05 - 1.09) | <0.001 |  | 1.06 (1.03 - 1.08) | <0.001 |
| T 1 | 1 |  |  |  | <0.001 |  |  | <0.001 |  |  | <0.001 |
| T 2 | 2.35 (1.44 - 3.82) | <0.001 |  | 2.09 (1.24 - 3.54) | 0.006 |  | 1.99 (1.17 - 3.41) | 0.012 |  | 1.68 (0.83 - 3.40) | 0.148 |
| T 3 | 8.35 (5.16 - 13.52) | <0.001 |  | 6.73 (3.93 - 11.51) | <0.001 |  | 6.37 (3.68 - 11.04) | <0.001 |  | 5.66 (2.85 - 11.27) | <0.001 |

**Model 1** Unadjusted

**Model 2** adjusted for age, sex, systolic blood pressure, diastolic blood pressure, smoking, drinking, body mass index, dysphagia.

**Model 3** adjusted for age, sex, systolic blood pressure, diastolic blood pressure, smoking, drinking, body mass index, dysphagiax, hypertension, diabetes, hyperlipidemia, atrial fibrillation.

**Model 4** adjusted for age, sex, systolic blood pressure, diastolic blood pressure, smoking, drinking, body mass index, dysphagia, hypertension, diabetes, hyperlipidemia, atrial fibrillation, red blood cell count, white blood cell count, platelet count, D-dimer.

OR, odds ratio; CI, confidence interval.

Supplementary table 5. Subgroup analysis of the association between the class of dynamic Wells score with stroke-associated pneumonia

| Variable | Class 2 vs. Class 1 | |  | Class 3 vs. Class 1 | |  | Class 4 vs. Class 1 | | *P* for interaction |
| --- | --- | --- | --- | --- | --- | --- | --- | --- | --- |
|  | OR (95% CI） | *P*-value |  | OR (95% CI） | *P*-value |  | OR (95% CI） | *P*-value |  |
| Age, years |  |  |  |  |  |  |  |  |  |
| ≤67 | 3.98 (2.08 - 7.62) | <0.001 |  | 9.42 (4.47 - 19.84) | <0.001 |  | 14.49 (5.26 - 39.91) | <0.001 | 0.925 |
| ＞67 | 2.31 (1.11 - 4.81) | 0.026 |  | 8.89 (4.14 - 19.09) | <0.001 |  | 26.29 (12.45 - 55.53) | <0.001 |  |
| Sex |  |  |  |  |  |  |  |  |  |
| Male | 3.41 (1.82 - 6.32) | <0.001 |  | 8.32 (4.11 - 16.86) | <0.001 |  | 30.56 (15.30 - 61.04) | <0.001 | 0.415 |
| Female | 2.89 (1.32 - 6.33) | 0.008 |  | 11.18 (4.95 - 25.24) | <0.001 |  | 28.99 (11.04 - 76.15) | <0.001 |  |
| Smoking |  |  |  |  |  |  |  |  |  |
| No | 3.35 (1.78 - 6.31) | <0.001 |  | 10.37 (5.24 - 20.53) | <0.001 |  | 28.05 (13.66 - 57.60) | <0.001 | 0.713 |
| Yes | 2.96 (1.38 - 6.31) | 0.005 |  | 8.83 (3.80 - 20.53) | <0.001 |  | 31.96 (13.03 - 78.38) | <0.001 |  |
| Drinking |  |  |  |  |  |  |  |  |  |
| No | 4.13 (2.20 - 7.74) | <0.001 |  | 12.13 (6.21 - 23.71) | <0.001 |  | 44.55 (21.68 - 91.54) | <0.001 | 0.151 |
| Yes | 2.13 (0.96 - 4.71) | 0.062 |  | 7.47 (2.98 - 18.73) | <0.001 |  | 13.43 (5.33 - 33.81) | <0.001 |  |
| Hypertension |  |  |  |  |  |  |  |  |  |
| No | 4.08 (1.96 - 8.51) | <0.001 |  | 14.70 (6.16 - 35.10) | <0.001 |  | 27.22 (11.28 - 65.67) | <0.001 | 0.393 |
| Yes | 2.66 (1.39 - 5.09) | 0.003 |  | 8.20 (4.16 - 16.15) | <0.001 |  | 31.28 (15.23 - 65.60) | <0.001 |  |
| Diabetes |  |  |  |  |  |  |  |  |  |
| No | 3.60 (2.07 - 6.29) | <0.001 |  | 12.10 (6.56 - 22.32) | <0.001 |  | 27.83 (14.61 - 52.99) | <0.001 | 0.195 |
| Yes | 2.21 (0.82 - 6.00) | 0.119 |  | 5.33 (1.82 - 15.59) | 0.002 |  | 35.43 (11.38 - 110.31) | <0.001 |  |
| Hyperlipidemia |  |  |  |  |  |  |  |  |  |
| No | 3.49 (2.09 - 5.83) | <0.001 |  | 9.72 (5.60 - 16.86) | <0.001 |  | 31.60 (17.54 - 56.92) | <0.001 | 0.145 |
| Yes | 1.60 (0.34 - 7.44) | 0.549 |  | 16.00 (2.00 - 127.93) | 0.009 |  | 13.33 (2.06 - 86.34) | 0.007 |  |
| Atrial fibrillation |  |  |  |  |  |  |  |  |  |
| No | 3.17 (1.79 - 5.61) | <0.001 |  | 8.20 (4.33 - 15.53) | <0.001 |  | 30.52 (14.68 - 63.42) | <0.001 | 0.158 |
| Yes | 3.17 (1.79 - 5.61) | <0.001 |  | 8.20 (4.33 - 15.53) | <0.001 |  | 30.52 (14.68 - 63.42) | <0.001 |  |
| WBC, 10^9^/L |  |  |  |  |  |  |  |  |  |
| ≤7.2 | 3.19 (1.48 - 6.87) | 0.003 |  | 8.74 (3.55 - 21.56) | <0.001 |  | 58.65 (24.36 - 141.22) | <0.001 | 0.065 |
| ＞7.2 | 2.78 (1.46 - 5.32) | 0.002 |  | 6.78 (3.43 - 13.42) | <0.001 |  | 13.87 (6.63 - 29.02) | <0.001 |  |
| PLT, 10^9^/L |  |  |  |  |  |  |  |  |  |
| ≤175 | 2.69 (1.37 - 5.29) | 0.004 |  | 8.04 (3.73 -17.35) | <0.001 |  | 31.96 (14.66 - 69.68) | <0.001 | 0..555 |
| ＞175 | 3.82 (1.90 - 7.70) | <0.001 |  | 10.85 (5.17 - 22.79) | <0.001 |  | 25.22 (11.22 - 56.71) | <0.001 |  |
| RBC, 10^12^/L |  |  |  |  |  |  |  |  |  |
| ≤4.5 | 2.77 (1.38 - 5.56) | 0.004 |  | 13.08 (6.08 - 28.13) | <0.001 |  | 29.44 (13.76 - 63.02) | <0.001 | 0.192 |
| ＞4.5 | 3.64 (1.85 - 7.18) | <0.001 |  | 6.74 (3.17 - 14.30) | <0.001 |  | 25.39 (10.94 - 58.91) | <0.001 |  |
| A^2^DS^2^ score |  |  |  |  |  |  |  |  |  |
| ≤4 | 2.01 (1.12 - 3.62) | 0.020 |  | 3.74 (1.82 - 7.70) | <0.001 |  | 12.22 (5.28 - 28.33) | <0.001 | 0.874 |
| ＞4 | 5.16 (1.79 - 14.84 ) | 0.002 |  | 16.14 (5.37 - 48.49) | <0.001 |  | 27.69 (9.42 - 81.37) | <0.001 |  |

WBC, white blood cell count; PLT, platelet count; RBC, red blood cell count.

Supplementary table 6. Subgroup analysis of the association between the cumulative Wells score with stroke-associated pneumonia

| Variable | T 2 vs. T 1 | |  | T 3 vs. T 1 | | *P* for interaction |
| --- | --- | --- | --- | --- | --- | --- |
|  | OR (95% CI） | *P*-value |  | OR (95% CI） | *P*-value |  |
| Age, years |  |  |  |  |  |  |
| ≤67 | 3.81 (1.96 - 7.43) | <0.001 |  | 10.36 (5.30 - 20.24) | <0.001 | 0.843 |
| ＞67 | 2.37 (1.30 - 4.30) | 0.005 |  | 8.72 (4.79 - 15.89) | <0.001 |  |
| Sex |  |  |  |  |  |  |
| Male | 3.68 (2.09 - 6.48) | <0.001 |  | 11.78 (6.70 - 20.72) | <0.001 | 0.718 |
| Female | 2.67 (1.33 - 5.35) | 0.006 |  | 9.54 (4.75 - 19.14) | <0.001 |  |
| Smoking |  |  |  |  |  |  |
| No | 3.59 (2.03 - 6.38) | <0.001 |  | 11.28 (6.34 - 20.07) | <0.001 | 0.901 |
| Yes | 2.74 (1.38 - 5.45) | 0.004 |  | 10.20 (5.18 - 20.09) | <0.001 |  |
| Drinking |  |  |  |  |  |  |
| No | 4.39 (2.52 - 7.64) | <0.001 |  | 13.21 (7.56 - 23.07) | <0.001 | 0.387 |
| Yes | 1.74 (0.81 - 3.76) | 0.156 |  | 7.80 (3.84 - 16.66) | <0.001 |  |
| Hypertension |  |  |  |  |  |  |
| No | 3.80 (1.95 - 7.70) | <0.001 |  | 15.09 (7.37 - 30.92) | <0.001 | 0.271 |
| Yes | 2.90 (1.65 - 5.11) | <0.001 |  | 9.05 (5.20 - 15.78) | <0.001 |  |
| Diabetes |  |  |  |  |  |  |
| No | 3.87 (2.32 - 6.45) | <0.001 |  | 12.11 (7.27 - 20.16) | <0.001 | 0.537 |
| Yes | 2.01 (0.86 - 4.74) | 0.109 |  | 7.98 (3.39 - 18.82) | <0.001 |  |
| Hyperlipidemia |  |  |  |  |  |  |
| No | 3.53 (2.22 - 5.62) | <0.001 |  | 11.38 (7.19 - 18.01) | <0.001 | 0.447 |
| Yes | 1.75 (0.45 - 6.77) | 0.417 |  | 6.00 (1.27 - 28.25) | 0.023 |  |
| Atrial fibrillation |  |  |  |  |  |  |
| No | 2.77 (1.64 - 4.71) | <0.001 |  | 8.63 (5.10 - 14.61) | <0.001 | 0.898 |
| Yes | 2.73 (1.14 - 6.57) | 0.024 |  | 8.57 (3.56 - 20.66) | <0.001 |  |
| WBC, 10^9^/L |  |  |  |  |  |  |
| ≤7.2 | 3.39 (1.69 - 6.79) | <0.001 |  | 14.11 (7.18 - 27.71) | <0.001 | 0.114 |
| ＞7.2 | 2.58 (1.44 - 4.64) | 0.002 |  | 7.04 (3.90 - 12.72) | <0.001 |  |
| PLT, 10^9^/L |  |  |  |  |  |  |
| ≤175 | 2.78 (1.51 - 5.14) | 0.001 |  | 10.56 (5.72 - 19.49) | <0.001 | 0.880 |
| ＞175 | 3.74 (1.99 - 7.03) | <0.001 |  | 10.55 (5.63 - 19.79) | <0.001 |  |
| RBC, 10^12^/L |  |  |  |  |  |  |
| ≤4.5 | 2.98 (1.67 - 5.32) | <0.001 |  | 9.61 (5.31 - 17.39) | <0.001 | 0.645 |
| ＞4.5 | 3.47 (1.75 - 6.87) | <0.001 |  | 11.93 (6.13 - 23.23) | <0.001 |  |
| A^2^DS^2^ score |  |  |  |  |  |  |
| ≤4 | 2.61 (1.38 - 4.60) | 0.020 |  | 4.12 (2.19 - 7.74) | <0.001 | 0.099 |
| ＞4 | 2.50 (1.15 - 5.45) | 0.021 |  | 8.56 (3.99 - 18.39) | <0.001 |  |

WBC, white blood cell count; PLT, platelet count; RBC, red blood cell count.
